# The clinical and genetic features in a cohort of mainland Chinese patients with thyrotoxic periodic paralysis

**DOI:** 10.1186/s12883-015-0290-8

**Published:** 2015-03-21

**Authors:** Xiaobing Li, Sheng Yao, Yining Xiang, Xiaolei Zhang, Xiangbing Wu, Laimin Luo, Haihua Huang, Min Zhu, Hui Wan, Daojun Hong

**Affiliations:** Department of Emergency, The First Affiliated Hospital of Nanchang University, Nanchang, China; Department of Neurology, The Navy General Hospital of China, Beijing, China; Department of Pathology, The Affiliated Hospital Guiyang Medical College, Guiyang, China; Department of Neurology, The People Hospital of Shanxi Province, Taiyuan, China; Department of Neurology, The Affiliated Hospital of Jiujiang College, Jiujiang, China; Department of Nephrology, The First Affiliated Hospital of Nanchang University, Nanchang, China; Department of Endocrinology, The First Affiliated Hospital of Nanchang University, Nanchang, China; Department of Neurology, The First Affiliated Hospital of Nanchang University, 17# Yong Wai Zheng Street, Nanchang, PRC

**Keywords:** Thyrotoxic periodic paralysis, Hyperthyroidism, *KCNJ18* gene, *KCNJ2* gene, Polymorphism

## Abstract

**Background:**

Thyrotoxic periodic paralysis (TPP) is a life-threatening channelopathy manifesting as recurrent episodes of hypokalemia and muscle weakness in the presence of hyperthyroidism. Recent findings indicate defects of inward rectifying K+ (Kir) channels are associated with some TPP patients. The associations are not only found in Caucasian population (mainly Brazilian), but also in Singaporean population. However, potential genetic risk factors for mainland Chinese patients, the largest group of TPP cases in the world, have been largely unexplored.

**Methods:**

Samples of DNA from 127 individuals with TPP and 102 hyperthyroidism male controls self-reported as mainland Chinese were collected from 5 clinical centers from Jan 2011 to Jan 2014. The *KCNJ2* gene, *KCNJ18* gene, as well as loci polymorphisms (rs623011and rs312691) at 17q24.3 were directly sequenced in TPP patients and controls. Clinical data were summarized from TPP participants for genotype/phenotype correlations.

**Results:**

3.1% of TPP cases harbored *KCNJ18* gene mutations in mainland Chinese patients. Patients with *KCNJ18* mutation had shorter attack duration, higher prevalence of muscle soreness and weakness recurrence than patients without *KCNJ18* mutation. The alleles at 17q24.3 (rs623011and rs312691) were more common in patients with TPP than in controls, and therefore were significant risk factors for TPP (odds ratio, 11.94 and 10.57; 95% CI, 5.93-24.05 and 5.48-20.40; P = 1.81 × 10^−14^ and 1.07 × 10^−14^ respectively).

**Conclusions:**

This study demonstrates that the *KCNJ18* variants are only responsible for a small proportion of TPP patients in mainland China. There are significant clinical differences between patients with *KCNJ18* mutations and patients without *KCNJ18* mutations. In addition, the rs623011and rs312691 loci are significantly associated with TPP patients in mainland China, and highlight the Kir2.1 channel as a causative target in TPP.

**Electronic supplementary material:**

The online version of this article (doi:10.1186/s12883-015-0290-8) contains supplementary material, which is available to authorized users.

## Background

Thyrotoxic periodic paralysis (TPP) is a disorder manifesting as recurrent episodes of hypokalemia and muscle weakness in the presence of hyperthyroidism. The condition may be life-threatening if breathing muscle weakness leads to breathing failure, or if the low potassium levels cause cardiac arrhythmias [1,2]. TPP is more prevalent in Asian populations, especially in males of Chinese, Japanese, Vietnamese, Filipino, and Korean descent [3,4]. In Chinese populations, TPP occurs in up to 13% of male thyrotoxic patients, while women are predominantly affected by hyperthyroidism in the general population [3,5]. The high incidence of TPP among Asian people suggests that the basic defects may be genetically determined [6].

Although the mechanism of TPP remains largely uncertain, a few risk factors have been identified, such as carbohydrate loads and rest following exercise [7]. When the symptoms of thyrotoxicosis are separated from the clinical picture, many features of this disease are similar to those described in familial hypokalemic periodic paralysis [8]. The condition has been linked to mutations in genes that code for certain ion channels that transport electrolytes across cell membranes [9]. So TPP is considered as an endocrine channelopathy with genetic background [10]. Recent findings indicate that defects of the skeletal muscle-specific inward rectifying K+ (Kir) channel, Kir2.6, encoded by the *KCNJ18* gene, is associate with a proportion of TPP patients mainly from the United States, Brazil, France and Singapore [11]. Other important developments are that gene polymorphisms (rs623011and rs312691) at 17q24.3 may affect the expression of *KCNJ2* gene (encoding Kir2.1) in Hong Kong and Thai populations [12,13]. In mainland China, the largest group of patients with TPP in the world exists [14]; however, the clinical and genetic features are seldom explored in these patients. In our study, we aimed to determine whether mutations in *KCNJ2* and *KCNJ18* exist in mainland Chinese patients, and assess whether loci polymorphisms (rs623011and rs312691) at 17q24.3 are associated with mainland Chinese patients.

## Methods

### Subjects

One hundred and twenty-seven Chinese patients with TPP were collected in five clinical centers (The First Affiliated Hospital of Nanchang University, Navy General Hospital of China, Affiliated Hospital Guiyang Medical College, People Hospital of Shanxi Province, and Affiliated Hospital of Jiujiang College) from January 2011 to January 2014. Diagnostic criteria for TPP include: (1) acute limb paralysis with lower motor neuron origin; (2) blood potassium concentration <3.5 mmol/L; (3) hyperthyroidism supported by laboratory tests; (4) excluding familial hypokalemic periodic paralysis and hypokalemic periodic paralysis caused by other causes. All subjects were examined and interviewed by at least two clinicians (Li X., Yao S., Xiang Y., Zhang X., Wu X., Wan H., and Hong D.), and then all of medical data uniformly reanalyzed by Dr. Li and Dr. Hong. In order to avoid the deviation of diagnosis, all patients must first meet the diagnostic criteria 1 and 2, and then accord with the diagnostic criteria 3 and 4. Controls of 102 hyperthyroid male patients without episodic weakness during their hyperthyroid states were collected from the First Affiliated Hospital of Nanchang University. All cases and controls self-reported as mainland Chinese were male and their etiology of hyperthyroidism was Graves’ disease. This study was approved by the ethics committee of the First Affiliated Hospital of Nanchang University. All participants gave their written informed consent in compliance with the Chinese bioethics laws as well as the Declaration of Helsinki.

### Genetic screening

Genomic DNA was extracted form peripheral blood of TPP cases and controls. Coding exons of the *KCNJ2* and *KCNJ18* genes were amplified using polymerase chain reaction (PCR) with intronic primers. The primers for amplification of the *KCNJ2* gene (NM000891.2) were F1: 5′GCTCCCAGAGACACCCATC3′, R1: 5′CATGACTGCGCCAATGATGA3′; and F2: 5′GACCCAGACAACCATAGGCT3′, R2: 5′CCCATCTTGACCAGTACCGT3′. The primer for the *KCNJ18* gene (NG033093) was F: 5′ATGCTGTCCTCTCTGTTCC3′ and R: 5′GGGCCTCTCCCCGGCCA3′. The primers for rs623011 and rs312691 were F: 5′TCAGTCAACCACAAACCACA3′, R: 5′CCAGCCAAGGAGACAACAAT3′ and F: 5′TCATGCCTGACCTGTGTACA3′, R: 5′AGCACTGAGAAGAGGTTGGG3′ respectively. After purification, PCR products were directly sequenced with an ABI 3730 DNA analyzer (Applied Biosystems, Inc., CA, USA). To exclude PCR errors, all nucleotide variations were sequenced in reverse. To exclude the possibility that *KCNJ18* mutations represented polymorphisms, identical genomic fragments from 200 healthy Chinese controls were examined for the novel mutations.

### Statistical analysis

The association of the different clinical variables was analyzed by univariate analysis. Continuous variables were expressed as mean ± SD and were analyzed by t’-test. Categorical variables were expressed as absolute number and proportion of cases and were compared by two-tail Fisher’s exact test. For genotype study, the allele and genotype distributions of TPP patients and controls were compared and evaluated in allelic, dominant and recessive inheritance models by two-tail Fisher’s exact test. Odds ratios (OR) and confidence intervals (CI) were calculated using the non-risk genotype (12 + 22) as reference. A value of *P* < 0.05 was considered statistically significant. All statistical studies were performed by SPSS 13.0 software.

## Results

### *KCNJ18* gene mutation

In the *KCNJ18* gene screening, genetic analysis revealed 4 patients with heterozygous point mutations in 127 TPP patients (4/127, 3.1%) (Additional file 1: Figure S1). One nonsense variant was at c.376C > T, which introduced a stop codon (p.Q126X). Two other novel missense mutations were c.1079A > C that caused threonine substitution for lysine at residue 360 (p.K360T); and c.1162G > A that resulted in a replacement of glutamate with lysine at residue 388 (p.E388K). Substitution p.K360T is predicted to affect protein function with a score of 0.00 in Sorting Intolerant From Tolerant (SIFT) software [15] and a score of 0.988 in Polyphen2 software [16]. Substitution p.E388K is predicted to affect protein function with a score of 0.03 in SIFT software and a score of 0.728 in Polyphen2 software. Blast software revealed that the two mutant residues had high evolutional conservation in Kir2.x protein family (Additional file 1: Figure S2 and S3) [17]. We also identified a variant (c.598G > C) leading to the change of alanine to proline at residue 200 (p.A200P) that was described in a Taiwanese with sporadic periodic paralysis [18]. The novel mutations were not identified in 200 healthy controls of Chinese descent.

### *KCNJ2* gene related mutations

No genetic variants were identified in the *KCNJ2* gene in this cohort of TPP patients. However, the genetic polymorphisms of rs312691 and rs623011 nearby the *KCNJ2* gene showed significant associations with TPP patients from five clinical centers (Table 1) (Additional file 1: Table S1 and S2). In rs623011, A is the minor allele (0.488 in Han Chinese in Beijing, China (CHB)) [19]. The allele frequency increased to 0.772 (196/254) in TPP patients, while A allele frequency was 0.461 (94/204) in hyperthyroid controls. In rs312691, C is minor allele (0.463 in CHB) [19]. The allele frequency increased to 0.799 (203/254) in TPP patients, while C allele frequency was 0.471 (96/204) in hyperthyroid controls. The polymorphisms had great associations with TPP occurrence, but there are no significant differences between clinical phenotype and genotype. Interestingly, the four patients with *KCNJ18* mutations carried A/G or G/G at rs623011, and C/T or T/T at rs312691, while no homogenous minor genotypes (A/A and C/C) were identified at the two alleles.

**Table 1:** **Genotype associations of rs623011 and rs312691 with TPP in mainland Chinese male patients**

	**Case**					**Control**				**P-value**				
	**Allele (1/2)**	**11**	**12**	**22**	**Frequency of risk allele (1)**	**11**	**12**	**22**	**Frequency of risk allele (1)**	**1vs2**	**11vs12 + 22**	**11 + 12vs22**	**Odds ratio**	**95% confidence interval**
rs623011	A/G	78	40	9	0.772	12	70	20	0.461	9.88 × 10^−12^	1.81 × 10^−14^	5.27 × 10^−3^	11.94	5.93-24.05
rs312691	C/T	82	39	6	0.799	15	66	21	0.471	2.64 × 10^−13^	1.07 × 10^−14^	3.08 × 10^−4^	10.57	5.48-20.40

Odds ratios and confidence intervals were calculated using the non-risk genotype (12 + 22) as reference.

### Clinical features of TPP

Four sporadic male patients harbored *KCNJ18* gene mutations (Table 2). The age at onset ranged from 19 to 25 years old (mean 21.75 ± 2.75). All subjects suffered from severe limbs weakness when getting up in the morning. In addition, three out of four patients complained of muscle soreness or limb numbness. The course of disease lasted for 2–8 (mean 4.8 ± 2.8) hours. Three patients had strenuous exercise before the attacks. Serum potassium was 1.6-2.4 mmol/L (normal reference 3.5-5.1 mmol/L) without abnormalities of serum magnesium and phosphorus. Serum creatine kinase (CK) of all patients was moderately elevated to 1205-1710U/L (normal reference 20-170U/L). Electrocardiogram showed a typical u-wave without other abnormal electrophysiology. The diagnosis of hyperthyroidism was not made before the attacks of weakness; however, medical history suggested that 3 patients had weight loss; 2 patients had mild hand tremor; and 1 patient had mild goiter. Diagnosis of hyperthyroidism was made in 4 patients based on thyroid stimulating hormone (TSH) <0.03 mU/L (normal reference 0.35-4.95 mU/L), and increased level of free tetraiodothyronine and free triiodothyronine. Weakness was resolved after supplements of potassium. The symptom recurred in 3 patients during the treatment, and one of them had four episodes in the 13–28 months follow-up, however, weakness did not occur again after remission of hyperthyroidism.

**Table 2:** **The clinical manifestations of TPP patients with** ***KCNJ18*** **gene mutations**

**Case**	**Gender** **/** **age**	**Attack duration**	**Symptoms of weakness**	**Hyperthyroidism**	**Potassium mmol** **/** **L**	**CK IU** **/** **L**	**TSH uIU** **/** **ml**	**FT3 pg** **/** **ml**	**FT4 ng** **/** **dl**	***KCNJ18*** **mutation**
1	Male/19	2 h	Quadriplegia	Weight loss, goiter	1.9	2093	<0.03	8.2	3.5	p.Q126X
2	Male/20	6 h	Quadriplegia	Weight loss	2.4	1476	<0.03	12.3	5.8	p.A200P
3	Male/23	3 h	Quadriplegia	Tremor	2.1	1205	<0.03	7.6	5.1	p.K360T
4	Male/25	8 h	Quadriplegia	Weight loss	1.6	2310	<0.03	16.8	9.3	p. E388K

*Abbreviation: CK* creatine kinase, *TSH* Thyroid Stimulating Hormone, *FT3* free triiodothyronine, *FT4* free thyroxine.

Compared with patient with *KCNJ18* variants (Table 3), statistical analysis revealed TPP patients without *KCNJ18* variants had a longer weakness duration ranging from 1 hour to 6 days (mean 27.6 ± 11.6 hours). In addition, TPP patients without *KCNJ18* variants had lower prevalence of muscle soreness or limb numbness, and lower recurrence of muscle weakness. Except for the above clinical variables, other clinical variables including age at onset, quadriplegia, level of serum CK, and hyperthyroidism symptoms had no differences between patients with *KCNJ18* variants and without *KCNJ18* variants. The follow-up period (2–35 months) in patients without *KCNJ18* variants showed that episodic weakness did not recur after remission of hyperthyroidism.

**Table 3:** **Analysis of clinical variables in TPP patients between with** ***KCNJ18*** **mutation and without** ***KCNJ18*** **mutation**

**Variables**	**With** ***KCNJ18*** **mutations (n = 4)**	**Without** ***KCNJ18*** **mutations (n = 123)**	**P value**
Age	21.75 ± 2.75	25.23 ± 16.67	0.487
Sex (m/f)	4/0 (100%/0)	123/0 (100%/0)	-
Weakness duration (h)	4.8 ± 2.8	27.6 ± 11.6	0.006
Soreness or numbness	3 (75.0%)	21 (17.1%)	0.021
Quadriplegia	4 (100%)	66 (53.7%)	0.127
HyperCKmia	4 (100%)	75 (61.0%)	0.296
Hyperthyroidism symptoms			
Tremor	2 (50.0%)	47 (38.2%)	0.639
Weight loss	3 (75.0%)	30 (24.4%)	0.054
Goiter	1 (25.0%)	12 (9.8%)	0.354
Weakness recurrence	3 (75.0%)	26 (21.1%)	0.046

## Discussion

Although the detailed mechanism of TPP remains unclear, TPP has been considered as a kind of channelopathy disease, because its clinical pictures are very similar to those of familial or sporadic hypokalemic periodic paralysis [1-3,6]. A milestone progression was made by *Ryan DP.* and his colleagues in 2010 [11]. They found that the defects of Kir2.6 were responsible for partial TPP patients: 33.3% in Caucasian population (mainly Brazilian), 25.9% in Singaporeans, and 1.2% in Hong Kong people. In addition, 1.7% TPP patients from Taiwan were identified with *KCNJ18* variations, while mutations in *KCNJ18* were also identified in sporadic periodic paralysis (SPP). It suggested that both SPP and TPP had the same molecular basis [18]. Our systematic screening of 127 TPP cases found 3 novel and 1 previously reported heterozygous mutations in the *KCNJ18* gene, indicating 3.1% mainland Chinese TPP patients harbored these gene mutations, which is a little higher than those in Hong Kong and Taiwan, but significantly lower than found in Brazil and Singapore.

Since the first cases with *KCNJ18* mutations were reported in 2010, only 20 TPP cases with *KCNJ18* mutations had been described in the worldwide [11,18]. All of the patients plus our four cases were male individuals, while 2%-5% of TPP patients without *KCNJ18* mutation were female cases [5,6]. The episodic weakness in patients with *KCNJ18* mutation was commonly resolved within several hours, while the mean duration of our non-*KCNJ18* patients lasted for a longer period than those of patients with *KCNJ18* mutation. Meanwhile, we observed patients with *KCNJ18* mutation had a higher prevalence of muscle soreness and weakness recurrence than patients without *KCNJ18* mutation. At present, the total number of TPP patients with *KCNJ18* mutations is relatively small. In order to confirm the clinical phenotype of TPP patients with *KCNJ18* mutations, more cases should be analyzed and summarized in the future.

The *KCNJ18* gene encodes Kir2.6 protein that is a specific muscle subtype of Kir2.x family [11]. Kir2.6 can physically associate with Kir2.1 and Kir2.2 to form heterotetramers on sarcolemma. Through heterotetramerization of subunits, Kir2.6 may control Kir2.1 and Kir2.2 abundance on the muscle plasma membrane, thus providing a mechanism to fine tune electrical responses [20,21]. The *KCNJ18* gene contains a thyroid-responsive *cis* element within its promoter that may regulate gene transcription [22]. Under the high thyroid hormone, mutational *KCNJ18* transcripts express lots of abnormal Kir2.6, and thus a dominant negative trafficking phenomenon can lead to endoplasmic reticulum retention of Kir2.x subunits [21]. Thus, mutational Kir2.6 may alter a delicate protein abundance and balance of electrical activity in muscle. In this study, the nonsense mutation (p.Q126X) apparently will cause the loss of function in Kir2.6 channel owing to the truncated protein. The p.A200P mutation had been reported in a Taiwanese with sporadic periodic paralysis, and no whole-cell current was detected in the A200P mutant by *in vitro* electrophysiological study [18]. As for the other two novel mutations (p.K360T and p.E388K) with high evolutional conservation, the charge of amino acids will undergo great changes (i.e. from strong positive charge of lysine to neutral charge of threonine; from negative charge of glutamate to strong positive charge of lysine). Although no functional data were available on the two mutations from the literature, the SIFT software and Polyphen2 software predicted the mutations to be probably damaging. Meanwhile, *in vitro* study had showed that p.T354M and p.K366R (nearby p.K360T and p.E388K) produced small decrease in current density and longer time required for half-maximal current degradation respectively [11]. Although electrophysiological studies of p.K360T and p.E388K have not been done in our present study, the residues p.T354M and p.K366R are located in intracellular C-terminal of Kir2.6, which may affect downstream phyosphorylation of ion channel signal conduction [11,21].

Kir2.1 (encoded by *KCNJ2* gene) is highly expressed in skeletal and cardiac muscles. It is one of important components in the Kir2.x subfamily that are involved in the determination of muscle electrical physiology, such as resting membrane potential and cell excitability [20,23]. It is also known that Kir2.1 can physically integrate with any one of the other Kir2.x proteins, such as Kir2.2, Kir2.3, Kir2.4, and Kir2.6, to form heterotetramers on cell membrane [20]. Although we could not find any deleterious *KCNJ2* mutations in our TPP cases through direct sequencing, earlier studies showed that Kir2.1 mutations lead to Andersen-Tawil syndrome (ATS) manifesting as periodic paralysis, ventricular ectopy, and dysmorphic features [24]. Both ATS and TPP are characterized by periodic paralysis. Periodic paralysis occurs in ATS independent of blood potassium concentration, but periodic paralysis in TPP can occur only in the presence of hypokalemia and thyrotoxic state. Both rs623011 and rs312691 polymorphism are located at the 17q24.3 locus, ~75 kb and ~150 kb downstream of the *KCNJ2* gene respectively. Transcriptional regulatory elements of genes can be located at regions far away from the target transcriptional units [25]. In expression quantitative trait locus (eQTL) analysis, the loci variants might affect the expression of Kir2.1 and result in episodic weakness similar to one of the clinical characteristics in ATS patients [26].

## Conclusions

The mutations in *KCNJ18* are only responsible to a small proportion of Chinese patients with TPP. Some clinical symptoms (i.e. attack duration, muscle soreness, and weakness recurrence) present with significant differences between patients with *KCNJ18* mutations and patients without *KCNJ18* mutations. We also demonstrate that the genetic variants rs623011 and rs312691 at 17q24.3 locus are important risk factors for TPP patients in mainland China.

## Additional file

Additional file 1:
**The supplemental figures presented with the mutant chromatograms of the** ***KCNJ18*** **gene, and evolutional conservation of p.K360T and p.E388K respectively.** The supplemental tables showed genotype associations of rs623011 and rs312691in five different clinical centers respectively.

## Abbreviations

TPP: Thyrotoxic periodic paralysis
Kir: Inward rectifying K+
PCR: Polymerase chain reaction
OR: Odds ratios
CI: Confidence intervals
CK: Creatine kinase
TSH: Thyroid stimulating hormone
SPP: Sporadic periodic paralysis
AST: Andersen–Tawil syndrome
eQTL: Expression quantitative trait locus
