# Supplementary material for: The clinical and genetic features in a cohort of mainland Chinese patients with thyrotoxic periodic paralysis
Source: BMC Neurol. 2015 Mar 21;15:38. doi: 10.1186/s12883-015-0290-8 (PMC4371807; doi:10.1186/s12883-015-0290-8)
Supplement: Additional file 1: — The supplemental figures presented with the mutant chromatograms of the KCNJ18 gene, and evolutional conservation of p.K360T and p.E388K respectively. The supplemental tables showed genotype associations of rs623011 and rs312691in five different clinical centers respectively. [file 12883_2015_290_MOESM1_ESM.pdf]

Title page (additional file)

The clinical and genetic features in a cohort of mainland Chinese patients with thyrotoxic periodic paralysis

Xiaobing Li[1,8†], Sheng Yao[2†], Yining Xiang[3], Xiaolei Zhang[4], Xiangbing Wu[5], Laimin Luo[6], Haihua Huang[7], Min Zhu[8], Hui Wan[8], Daojun Hong[8*]

1 Department of Emergency, The First Affiliated Hospital of Nanchang University,

2 Department of Neurology, The Navy General Hospital of China, Beijing, China

3 Department of Pathology, The Affiliated Hospital Guiyang Medical College

4 Department of Neurology, The People Hospital of Shanxi Province

5 Department of Neurology, The Affiliated Hospital of Jiujiang College

6 Department of Nephrology, The First Affiliated Hospital of Nanchang University,

7 Department of Endocrinology, The First Affiliated Hospital of Nanchang University,

8 Department of Neurology, The First Affiliated Hospital of Nanchang University,

* The authors equally contributed to this article.

Corresponding author:

Daojun Hong MD, PhD,

E-mail: hongdaojun@hotmail.com

Department of Neurology, The First Affiliated Hospital of Nanchang University

17# Yong Wai Zheng Street, Nanchang, 330006, P.R.China.

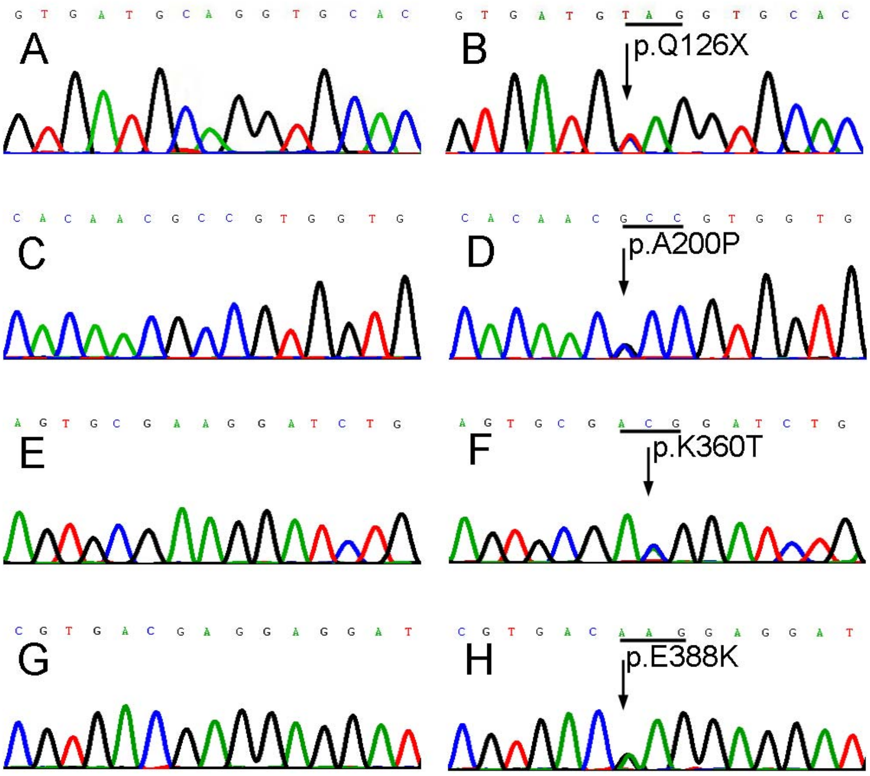

Figure 1. Genetic chromatogram of the *KCNJ18* gene. a nonsense mutation c.376C>T causing p.Q126X (B), A: control; a missense mutation c.598G>C causing p.A200P (D), C: control; a missense mutation 1079A>C causing p.K360T (F), E: control; a missense mutation c.1162G>A causing p.E388K (H), G: control.

```
Kir2.6 EVPSTPRCSAKDLVENKFLLP
Kir2.1 EVPNTPLCSARDLAEKKYILS
Kir2.2 EVPSTPRCSAKDLVENKFLLP
Kir2.3 EVAGTPCCSARELQESKITVL
Kir2.4 EVPGTPVCSAKELDERAEQAS
```

Figure 2. Blast software revealed that p.K360T mutation in Kir2.6 had highly evolutional conservation in Kir2.x protein family.

```
Kir2.6 CYENELAFLSRDEEDEADQ
Kir2.1 CYENEVALTSKEEDDSENG
Kir2.2 CYENELAFLSRDEEDEADG
Kir2.3 CYENELALMSQEEEEMEEE
Kir2.4 CYENELALSCCQEEDEDDE
```

Figure 3. Blast software revealed that p.E388K mutation in Kir2.6 had highly evolutional conservation in Kir2.x protein family.

Table 1. Genotype associations of rs623011 in mainland Chinese male patients with TPP from 5 different clinical centers

Clinical center	cases	allele(1/2)	11	12	22	frequency of risk allele(1)
The First Affiliated Hospital of Nanchang University	79	A/G	47	27	5	0.766
Navy General Hospital of China	21	A/G	13	6	2	0.762
Affiliated Hospital Guiyang Medical College	9	A/G	6	3	0	0.833
People Hospital of Shanxi province	7	A/G	4	2	1	0.714
Affiliated Hospital of Jiujiang College	11	A/G	8	2	1	0.818

Table 2. Genotype associations of rs312691 in mainland Chinese male patients with TPP from 5 different clinical centers

Clinical center	cases	allele(1/2)	11	12	22	frequency of risk allele(1)
The First Affiliated Hospital of Nanchang University	79	C/T	51	24	4	0.797
Navy General Hospital of China	21	C/T	13	7	1	0.786
Affiliated Hospital Guiyang Medical College	9	C/T	6	3	0	0.833
People Hospital of Shanxi province	7	C/T	4	3	0	0.786
Affiliated Hospital of Jiujiang College	11	C/T	8	2	1	0.818
